# Genome Sequence of *Rhizobium esperanzae* Type Strain CNPSo 668, Isolated from *Phaseolus vulgaris* Nodules in Mexico

**DOI:** 10.1128/genomeA.00935-17

**Published:** 2017-08-31

**Authors:** Luisa Caroline Ferraz Helene, Renan Augusto Ribeiro, Mariangela Hungria

**Affiliations:** a Embrapa Soja, Londrina, Brazil; b Universidade Estadual de Londrina (UEL), Department of Biochemistry and Biotechnology, Londrina, Brazil; c Coordenação de Aperfeiçoamento de Pessoal de Nível Superior (CAPES), Brasília, Brazil; d Conselho Nacional de Desenvolvimento Científico e Tecnológico (CNPq), Brasília, Brazil

## Abstract

*Rhizobium esperanzae* CNPSo 668^T^ is a nitrogen-fixing symbiont of *Phaseolus vulgaris* isolated from Mexican soils. Its genome is estimated at 6,294,057 bp, with 6,219 coding sequences (CDSs) showing higher similarity (92.9%) with *Rhizobium etli*. Three copies of the regulatory *nodD*, in addition to other nodulation genes, should define its host specificity.

## GENOME ANNOUNCEMENT

The common bean (*Phaseolus vulgaris* L.) is an important nitrogen-fixing legume with genetic origins in the Mesoamericas (main center) and the Andean regions. It establishes root-nodule symbioses with a variety of rhizobial species, but *Rhizobium etli* has been recognized as the main symbiont in genetic centers (1); however, we have previously shown that other rhizobial species coexist with the common bean in both centers (2, 3). Three strains (CNPSo 661, CNPSo 666, and CNPSo 668^T^) isolated from root nodules of the common bean in Mexico were positioned in a different cluster (1), and recent studies resulted in the description of a new species for this group, *Rhizobium esperanzae*, named after Esperanza Martínez-Romero, a brilliant rhizobiologist at the Center of Genomic Sciences, Cuernavaca, Mexico (4). Here we present the draft genome sequence of the type strain CNPSo 668 (= UMR 1320^T^ = Z87-8^T^ = LMG 30030^T^ = U 10001^T^).

Total DNA was extracted using the DNeasy blood and tissue kit (Qiagen) and processed on the MiSeq platform (Illumina) at Embrapa Soja. Paired-end reads obtained by shotgun sequencing allowed a genome coverage of 21-fold. The genome was assembled by the A5-miseq pipeline (*de novo* assembly) (5) and estimated at 6,294,057 bp, with 77 contigs and a G+C content of 61.1 mol%. The average nucleotide identity (ANI) with the closest species, *R. etli* CFN 42^T^, was 92.9%.

Sequences were submitted to RAST (6), and the annotation identified 6,219 coding sequences (CDSs), with 43% classified in 467 subsystems. Of the nonclassified CDSs, 1,861 were hypothetical and 1,715 were nonhypothetical. The major subsystems were of metabolism of carbohydrates (637 CDSs) and amino acids and derivatives (537 CDSs). Other interesting categories included 86 CDSs related to virulence, disease, and defense, most (76%) of which involved resistance to antibiotics and toxic compounds, 172 CDSs classified in a variety of stress response categories, and 135 CDSs for motility and chemotaxis. We found 10 *luxR* transcriptional regulators, with an *N*-acyl-l-homoserine lactone (AHL) synthase protein adjacent to an AHL-dependent transcriptional regulator, as well as CDSs related to type I, II, III, and IV secretion systems.

In relation to the symbiosis, the key gene responsible for starting the nodulation process is the regulatory gene *nodD* (7), and the genome of CNPSo 668^T^ encompasses three copies of *nodD*, showing the highest identities with *nodD1*, *nodD2*, and *nodD3* of *Rhizobium* sp. strain CCGE510. Interestingly, CCGE510 belongs to the large clade of *R. etli*, but it was isolated from the endangered species *Phaseolus albescens* in Mexico and reported as nitrogen-fixing ineffective with common beans (8). However, the remaining *nod* genes of CNPSo 668^T^, such as *nodC*, *nodI*, and *nodS*, showed higher identities with other symbionts of common bean of the large clade *R. etli-R. leguminosarum-R. phaseoli*. Therefore, the remaining *nod* genes of CNPSo 668^T^ should define the host range, supporting the hypothesis that differences have accumulated since the divergence of *Phaseolus* species (8).

### Accession number(s).

The whole-genome shotgun project has been deposited at DDBJ/EMBL/GenBank under the accession number SUBID SUB2473249, BioProject number PRJNA378648, BioSample number SAMN06555453, and accession number MXPU00000000. The version described in this paper is MXPU01000000.
